# Validated space radiation exposure predictions from earth to mars during Artemis-I

**DOI:** 10.1038/s41526-025-00459-y

**Published:** 2025-02-11

**Authors:** Tony C. Slaba, Shirin Rahmanian, Stuart George, Diego Laramore, John W. Norbury, Charles M. Werneth, Cary Zeitlin

**Affiliations:** 1 NASA Langley Research Center, Hampton, VA 23681 USA; 2 Analytical Mechanics Associates, Hampton, VA 23666 USA; 3 Space Radiation Analysis Group, NASA Johnson Space Center, Houston, TX 77058 USA; 4 Space Exploration and Mission Operations, Leidos Inc., Houston, TX 77598 USA

**Keywords:** Techniques and instrumentation, Biophysics

## Abstract

Accurate characterization of space radiation exposure is critical to assess and communicate multiple health risks for crewmembers participating in future exploration missions. A combination of models and on-board instruments are utilized to meet this requirement. In this work, computational models are evaluated against spaceflight measurements taken within the International Space Station, the Orion spacecraft, the BioSentinel CubeSat, and on the Martian surface. All calculations and measurements cover the exact same time period defined by the Artemis-I mission, and all model calculations were performed blind—without prior knowledge of the measurements. The models are shown to accurately characterize the absorbed dose-rate in highly complex and diverse shielding configurations in locations from Earth to Mars.

## Introduction

Artemis-I signaled the beginning of a new era in human spaceflight. The short mission, which launched on November 16, 2022 and ended on December 11, 2022, set in motion a broader vision of establishing permanent human presence on the moon under the Artemis program (https://www.nasa.gov/specials/artemis) and ultimately reaching Mars (https://www.nasa.gov/moontomarsarchitecture). Exposure to space radiation remains one of the most significant challenges limiting human exploration activities, as recognized by NASA^1^, international partners^2^, the National Academies of Science, Engineering, and Medicine^3^, the International Commission on Radiation Protection^4^, and the National Council on Radiation Protection^5^. Projected exposure levels for planned Artemis and Mars missions will lead to increased risks for numerous adverse health effects, including carcinogenesis, cardiovascular disease, and central nervous system decrements^1–5^.

To quantify these risks, the space radiation environment encountered by humans within habitable volumes in space must be accurately characterized. For Artemis and Mars missions, the primary focus will be on the radiation received beyond low Earth orbit (LEO) in the form of solar particle events (solar storms) and galactic cosmic rays (GCR). Solar storms give rise to intense bursts of energetic particles from the Sun that can last several hours or days. From a human risk perspective, the primary interest lies in the medium energy protons (<300 MeV) that can be accelerated in such events. If humans encounter a storm during extravehicular activities or surface operations without adequate shielding, whole body exposures can become elevated enough to initiate acute radiation syndrome responses and possibly death^6^. Fortunately, solar storm exposures behind shielding provided by human rated space vehicles are sufficiently mitigated through design optimization and operational countermeasures, such as storm shelter construction, to avoid these imminent health risks^6^.

GCR, on the other hand, encompass a broad spectrum of highly penetrating particles covering elements up to Ni and beyond with energies up to 10^13 ^MeV. The GCR environment represents a continuous low dose-rate exposure, leading to appreciable total doses and multiple highly uncertain health consequences^1–5^. Shielding strategies are largely ineffective at appreciably reducing GCR exposures or risk, and validated model calculations show that too much shielding or poorly chosen shield materials can increase exposures due to nuclear interactions and associated secondary radiations such as neutrons and pions^7,8^. Accurately characterizing the full spectrum of particles and energies comprising the GCR induced field found within radiosensitive tissue sites of humans behind shielding is required to assess health risks for Artemis missions and beyond.

The Artemis-I mission marked a unique opportunity to evaluate the status of GCR modeling and measurement capabilities required to achieve broader exploration goals. During the mission (November 16, 2022–December 11, 2022), measurements of absorbed dose-rate in silicon were simultaneously being collected behind diverse and complex shield configurations at multiple destinations of interest to humans. These included measurements within the International Space Station (ISS) in LEO, the uncrewed Orion capsule in deep space, the BioSentinel CubeSat in deep space, and on the surface of Mars.

The exposure variation within a spacecraft at a given destination is also of interest, as crew will move continuously throughout the habitable volume and dynamically alter their accumulated dose. To address this point, multiple radiation detectors have been deployed within the interior of the ISS, providing a means to compare instrument readings and evaluate exposure differences caused by shielding variations at locations throughout the station^9,10^. During Artemis-I, detectors were operating on ISS in distinct locations within the US Lab and Cupola. Three detectors were collecting data on Orion at locations referred to as HPU, HSU1, HSU2. Comparison of these intravehicular readings demonstrates the sensitivity of measurement instruments to local shielding variation and provides a more rigorous test of computational models.

In this work, state-of-the-art space radiation environment, nuclear physics, and radiation transport models in use by NASA are combined and used to calculate GCR dose-rates for each of the aforementioned cases. Model calculations are compared to spaceflight measurements obtained with active charged particle detectors. Two aspects of this work are unique. First, never before have combined models been compared to spaceflight data associated with such diverse shielding configurations and in destinations covering LEO, deep space, and the surface of Mars *over the exact same time period*. Such a comparison provides an opportunity to validate models in multiple locations and shielding conditions under identical solar conditions. Second, all model calculations were performed blind—without having prior knowledge of the measured data values. The importance of this work is also twofold. First, the comparisons are comprehensive in nature, covering shielding masses from CubeSats (14 kg, BioSentinel) to space stations (~400,000 kg, ISS) and destinations from LEO to Mars. Second, and more importantly, this work demonstrates both measurement and modeling capabilities required for human risk quantification as we explore deep space, the lunar surface, and Mars.

## Results and discussion

### The space radiation environment

The space radiation environment is fundamentally different than any terrestrial source, making characterization of both the radiation field itself and subsequent health risks difficult and uncertain^11^. The ambient GCR environment beyond LEO includes nuclei from hydrogen to nickel that have been stripped of their electrons. Ions heavier than nickel are also present but in much lower magnitude and therefore make negligible contributions to absorbed dose and health risks for humans. The energy spectrum for each particle covers several orders of magnitude, but the peak fluence occurs near ~300 MeV/n. The overall GCR intensity is modulated by the heliospheric magnetic field (HMF) on an ~11 year cycle. During solar minimum, the HMF is at its weakest state, and GCR intensities are maximal. During solar maximum, the HMF is most intense, and GCR intensities are reduced, but the likelihood of episodic solar storms increases.

**Fig. 1:**
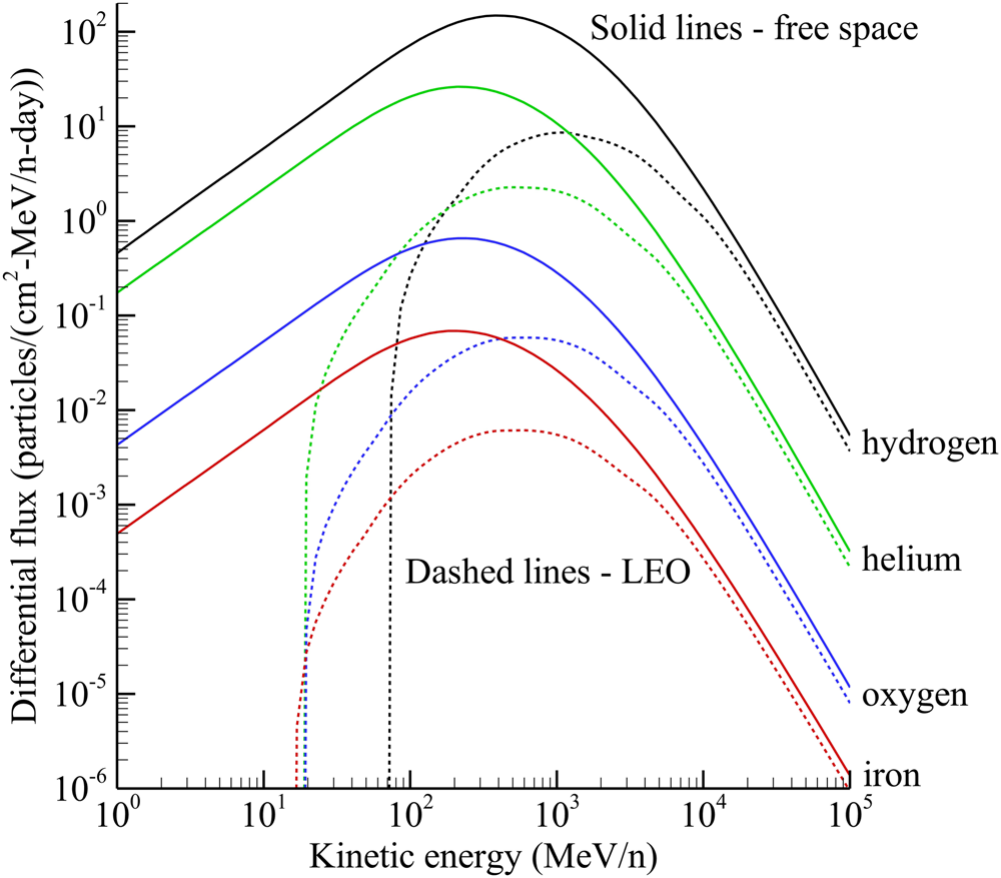
Selected particle flux energy spectra for the ambient GCR environment in free space (solid line) and ISS trajectory in LEO (dashed line). Both environments were calculated using the Artemis-I mission dates of November 16, 2022–December 11, 2022. Details of the models used to calculate these spectra can be found in the Methods section.

In Fig. 1, selected ions from the calculated ambient GCR environment at 1 Astronomical Unit (AU) for the Artemis-I mission time period are shown. Hydrogen and helium account for 89% and 10% of the particle flux, respectively. The remaining 1% consists of the ions with charge Z > 2. Although the flux intensities peak near ~300 MeV/n, as discussed, particle energies extend higher by several orders of magnitude. These more energetic ions are highly penetrating and capable of propagating through spacecraft shielding.

The ISS trajectory follows a circular orbit with an average altitude of 400–450 km, inclined 51.6^o^ from the equatorial plane. Radiation exposure on ISS is driven by geomagnetically modified GCR and repeated passes through the trapped proton belt within the South Atlantic Anomaly (SAA). The belts are populated with intense fluxes of medium energy protons able to penetrate the ISS mass shielding in certain locations and contribute roughly half of the total exposure^10^. Measurement and model validation of the belt exposures continues to be studied but is not the focus of this work. Emphasis is instead placed on the geomagnetically modified GCR exposure, providing contrast for GCR measurements and calculations at free space and Mars.

The GCR environment in LEO experienced by the ISS is attenuated due to the magnetosphere and the terrestrial shadow cast by the Earth. Both attenuating factors can be seen in Fig. 1. Attenuation in the energy spectra below 1 GeV/n for hydrogen and 500 MeV/n for heavier ions is a consequence of geomagnetic deflection for low-to-medium energy charged particles. The amount of geomagnetic shielding depends on the location and altitude of the spacecraft. Maximal deflection occurs near the equator. Conversely, the LEO GCR environment approaches free space conditions toward polar latitudes. This location dependence will be used in the next section to establish a smooth relationship between LEO and free space dose-rates.

The LEO GCR spectra shown in Fig. 1 are integrated over the ISS trajectory and therefore include geomagnetic attenuation levels from all latitudes between 0^o^ and 51.6^o^. The terrestrial shadow (blockage) provided by the Earth can be seen at the highest energies in Fig. 1, where the LEO spectra are 68% that of free space. The spectra from Fig. 1 are used as input to radiation transport codes to allow exposures behind shielding to be calculated.

### Radiation transport and shielding geometry

As the free space or LEO GCR environments encounter shielding, a multitude of atomic and nuclear interactions take place adding further complexity to the radiation environment. Atomic interactions reduce the velocity of charged particles, and nuclear interactions lead to fragmentation of incoming particles and target constituents. These fragmentation events yield secondary reaction products such as neutrons and pions capable of penetrating even farther through shielding.

Radiation transport codes are combined with nuclear and atomic physics models to describe these interactions, and realistic descriptions of the spacecraft mass shielding surrounding a detector or human are required to facilitate accurate intravehicular exposure calculations. The NASA deterministic radiation transport code, referred to herein as HZETRN2020 (High charge, Z, and Energy TRaNsport)^12–14^, is utilized in this work and has demonstrated the ability to analyze highly detailed mass shielding geometry for space radiation environments. Separate calculations were performed in this work with native nuclear physics models developed for HZETRN2020 and with a coupled nucleon/pion cross section database generated with Geant4^15^. Evaluating both sets of physics models in transport calculations provides additional context for comparisons to space measurements, as will be shown and discussed in the next subsection.

**Fig. 2:**
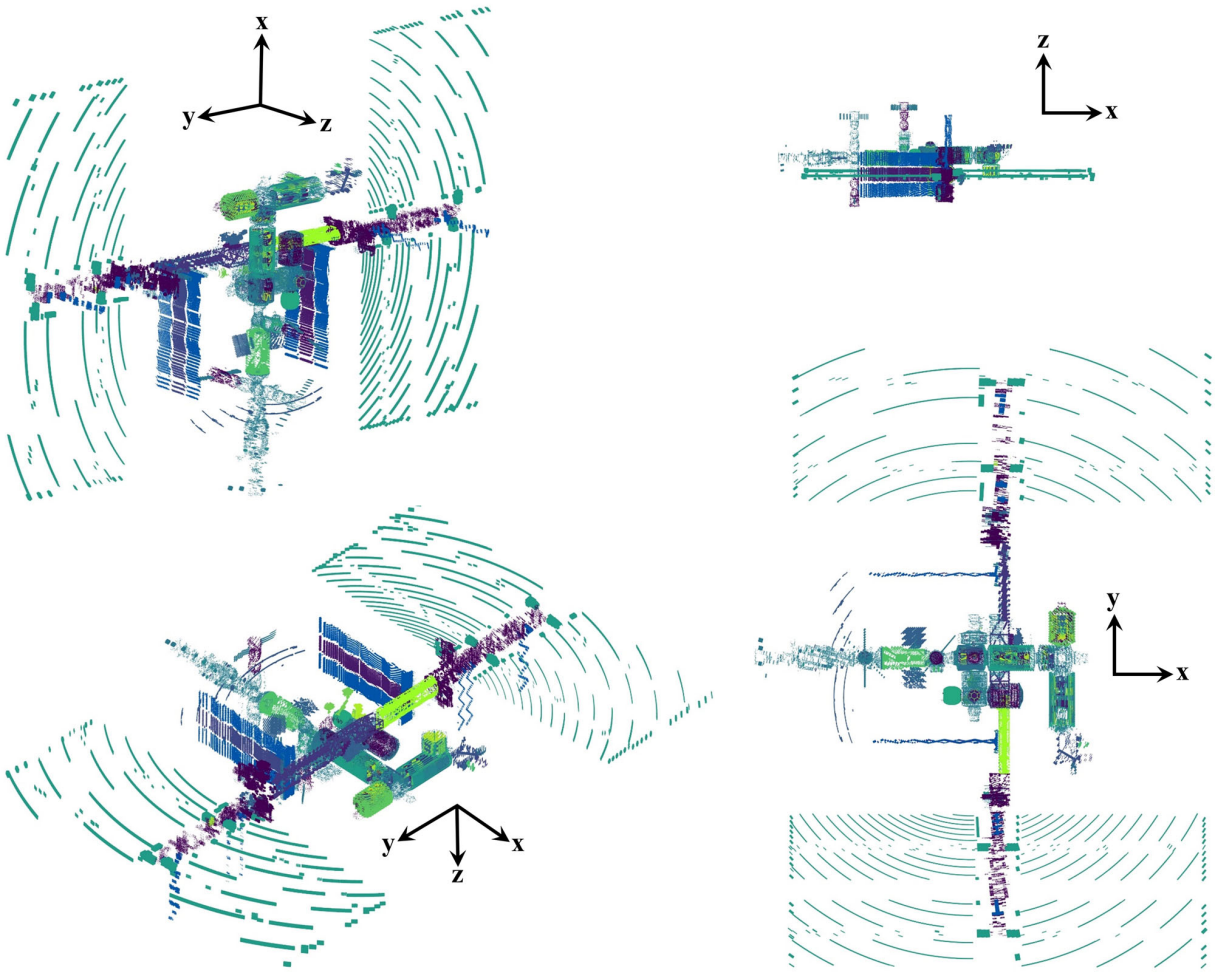
Scatter plot rendering of the ISS geometry used for model calculations; colors are used to identify unique objects retained in radiation transport analyses. A detailed CAD model of the ISS containing over 11,000 objects is ray-traced from a dosimeter location within the Cupola (observation module), and the ray-trace information is used as input into HZETRN2020. The scatter plot is a rendering of the geometry as interpreted and used by HZETRN2020 in transport analyses.

**Fig. 3:**
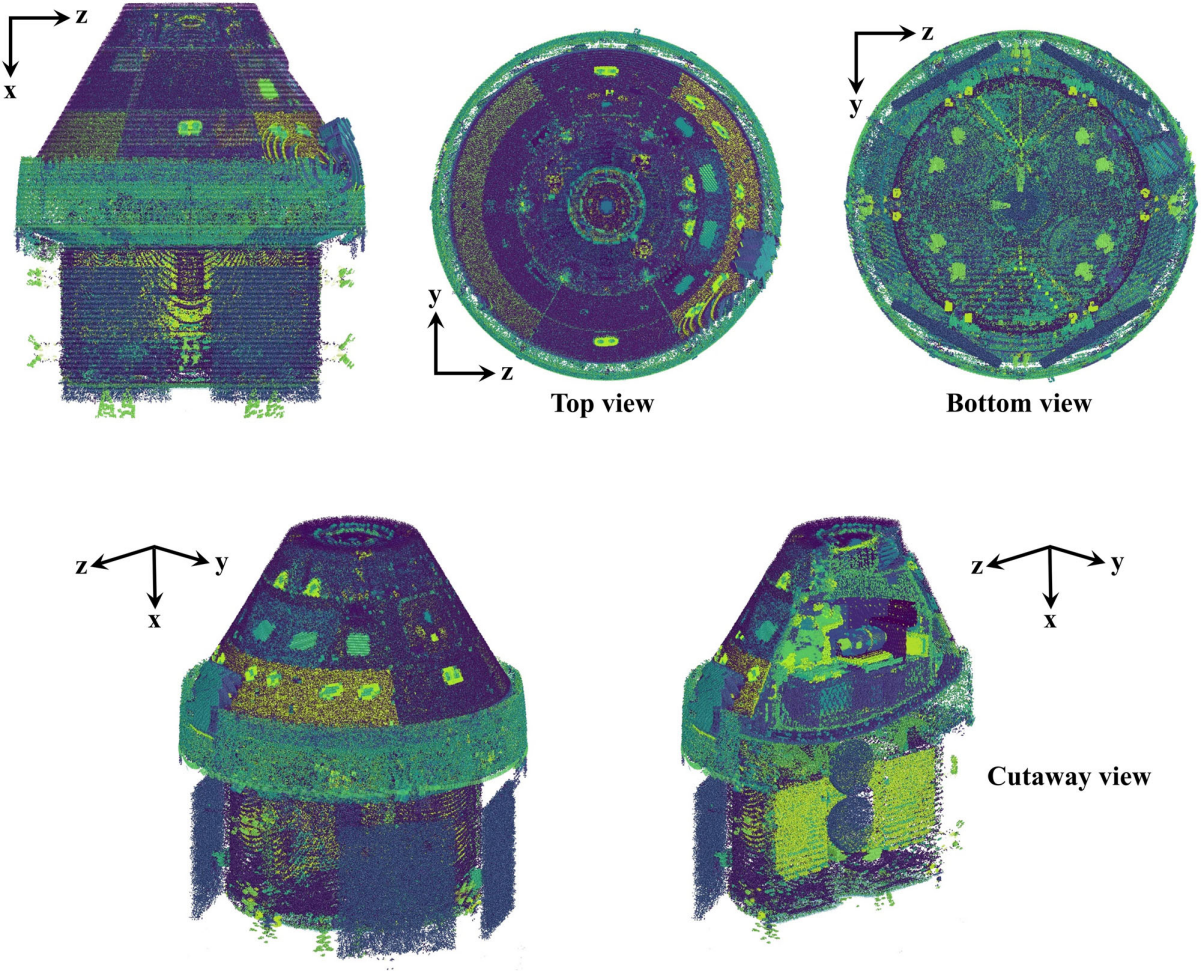
Scatter plot rendering of the Orion geometry used for model calculations; colors are used to identify unique objects retained in radiation transport analyses. A detailed CAD model of the Orion module containing over 20,000 objects is ray-traced from a dosimeter location within Orion, and the ray-trace information is used as input into HZETRN2020. The scatter plot is a rendering of the geometry as interpreted and used by HZETRN2020 in transport analyses.

The ISS and Orion geometries utilized in this study were developed in CAD software and contain over 11,000 and 23,000 parts, respectively. Figures 2 and 3 show the ISS and Orion geometries as interpreted by HZETRN2020 from the CAD information. The complexity, location, and shape of objects, regardless of size, is accurately retained for analysis in radiation transport calculations. For the BioSentinel CubeSat, a combinatorial geometry description (i.e. combinations of nested boxes and thin plates) was used, as demonstrated previously^16^. And in the case of the Mars surface, previously validated^17–19^ methods for representing the variable density carbon dioxide atmosphere and regolith surface were employed. Importantly, the vertical atmospheric column thickness of 21.4 g/cm^2^ measured by the Curiosity Rover Environmental Monitoring Station (REMS) sensor during the time period of the Artemis-I mission was retained in the geometry description.

**Fig. 4:**
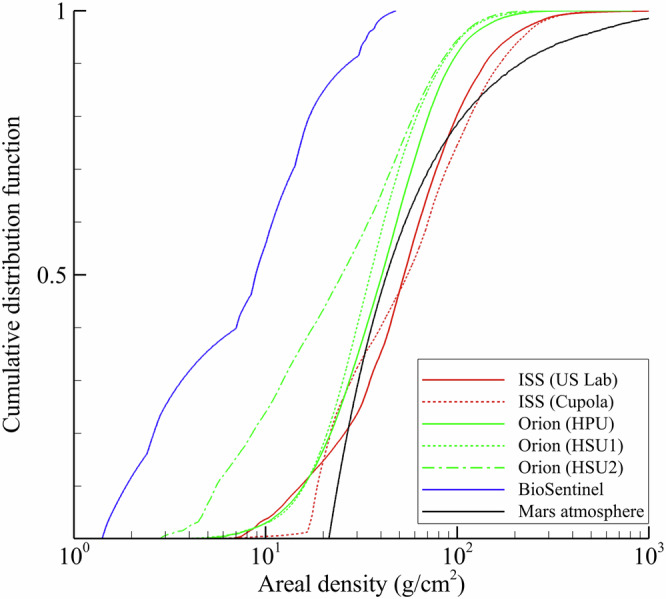
Mass shielding distributions for locations in the ISS, Orion, BioSentinel, and Mars surface. The distributions are calculated from ray-trace information and reflect the highly variable shielding surrounding a detector location.

The distribution of thicknesses for locations in ISS, Orion, BioSentinel, and on Mars is shown in Fig. 4. Representative values from the distributions are provided in Table 1. The ISS detector locations are more heavily shielded than all other vehicles considered herein, as expected. Shielding distributions for the US Lab and Cupola locations are quite similar though. On Orion, it can be seen that shielding levels decrease when moving from the HPU to HSU1 and HSU2. The BioSentinel shielding is the least massive of all geometries considered. Atmospheric shielding on Mars includes a notable minimum areal density of 21.4 g/cm^2^ associated with the vertical column thickness (i.e. looking straight up from the surface), as can be seen in Fig. 4. A broad distribution of thicknesses is observed as the zenith angle approaches the horizon, with maximum values exceeding 1000 g/cm^2^. The information regarding areal density distributions for these mass shielding geometries will be helpful to interpret modeling and measurement results presented in the next subsection. Collectively, it can be seen that geometry handling capabilities developed for HZETRN2020 are highly flexible and allow analysis through a wide range of shielding geometry scenarios of interest to human spaceflight.

**Table 1:** Representative areal densities (g/cm^2^) from the shielding distributions for ISS, Orion, BioSentinel, and Mars atmosphere

Vehicle	Detector	Aluminum shielding thickness (g/cm^2^)			
		Median	Mean	Minimum	Maximum
ISS	US lab	52.8	71.0	3.6	4796.3
	Cupola	55.0	76.8	1.2	2612.9
Orion	HPU	40.5	49.5	3.9	821.4
	HSU1	35.1	44.0	3.6	624.5
	HSU2	25.0	36.0	2.4	411.7
BioSentinel	BPD	8.9	11.3	1.4	48.6
Curiosity	MSLRAD	42.3	102.9	21.4	1234.5

### Comparison of model calculations to measurements

**Fig. 5:**
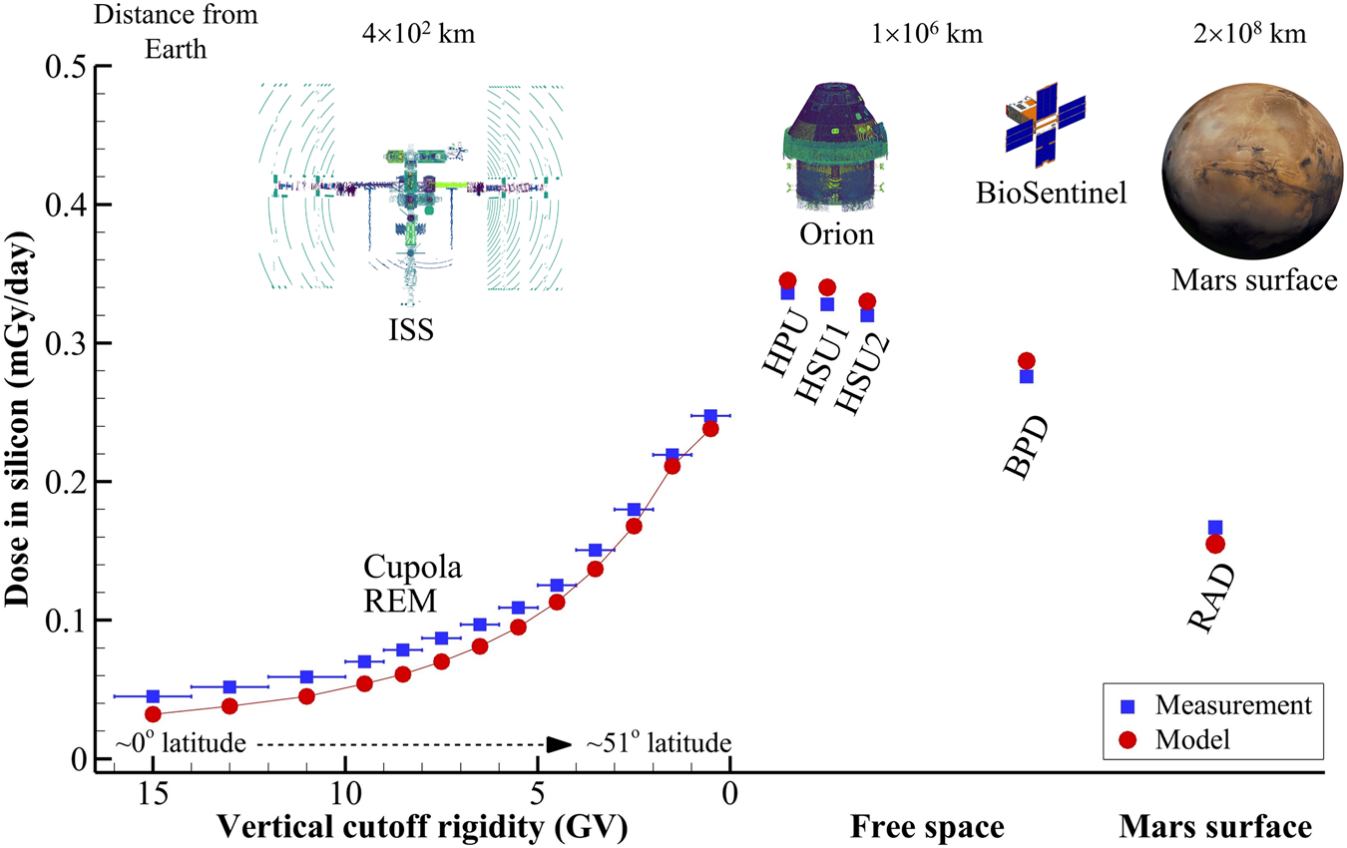
Comparison of model calculations to measurement data for the ISS, Orion module, BioSentinel CubeSat, and Mars surface during the Artemis-I mission. Approximate distances of each shielding geometry from the Earth are given at the top of the figure. Calculations and data for the ISS only include the GCR contributions. Exposure from trapped protons during passes through the South Atlantic Anomaly is not included in this comparison. ISS data are also binned according to vertical cutoff rigidity to show the trend associated with geomagnetic shielding.

A visual comparison of model-calculated absorbed dose-rates to measurements is provided in Fig. 5. Tabular data and relative differences between model and measurements are given in Tables 2 and 3. In Table 2, the ISS measurement data and model calculations both show increasing dose rates with decreasing vertical cutoff rigidity. Model calculations are well correlated with the measurements (correlation coefficient > 0.996) for both detector locations and both nuclear physics models, indicating that qualitative features of the LEO GCR environment as a function of vertical cutoff rigidity are being represented by the geomagnetic field model employed in this study.

**Table 2:** Comparison of measured silicon dose-rates (mGy/day) to model calculations in the ISS Cupola and US Lab as a function of vertical cutoff rigidity (GV) during the Artemis-I mission

Rigidity bin (GV)	Silicon dose-rate (mGy/day)					
	Cupola			US Lab		
	REM	Model	Model (G4)	RAD	Model	Model (G4)
0-1	0.248	0.238 (-4)	0.241 (-3)	0.269	0.239 (-11)	0.243 (-10)
1-2	0.219	0.211 (-4)	0.213 (-3)	0.244	0.210 (-14)	0.213 (-13)
2-3	0.180	0.168 (-7)	0.170 (-6)	0.201	0.166 (-18)	0.168 (-17)
3-4	0.151	0.137 (-9)	0.139 (-8)	0.171	0.134 (-21)	0.137 (-20)
4-5	0.125	0.113 (-10)	0.116 (-8)	0.145	0.110 (-24)	0.113 (-22)
5-6	0.109	0.095 (-13)	0.098 (-10)	0.125	0.092 (-27)	0.095 (-24)
6-7	0.097	0.081 (-17)	0.085 (-12)	0.112	0.078 (-31)	0.082 (-27)
7-8	0.087	0.070 (-20)	0.075 (-14)	0.101	0.067 (-34)	0.072 (-29)
8-9	0.078	0.061 (-22)	0.066 (-15)	0.092	0.058 (-37)	0.063 (-31)
9-10	0.070	0.054 (-23)	0.059 (-15)	0.082	0.051 (-38)	0.057 (-31)
10-12	0.059	0.045 (-23)	0.051 (-14)	0.069	0.043 (-39)	0.048 (-31)
12-14	0.052	0.038 (-27)	0.043 (-16)	0.061	0.036 (-41)	0.041 (-32)
>14	0.045	0.032 (-28)	0.038 (-16)	0.054	0.030 (-44)	0.035 (-34)

Values in parentheses are the relative difference (%) between model and measurement. Two sets of model calculations are provided. One set utilizes the native cross sections models developed for HZETRN2020, and the other set utilizes HZETRN2020 but with a coupled nucleon and pion interaction database generated from the Geant4 toolkit as described in the Methods section.

**Table 3:** Comparison of measured silicon dose-rates (mGy/day) to model calculations for ISS (Cupola and US lab), Orion (HPU, HSU1, HSU2), BioSentinel (BPD), and the surface of Mars (MSLRAD) during the Artemis-I mission

Location	Vehicle	Silicon dose-rate (mGy/day)			
		Detector	Measurement	Model	Model (G4)
LEO	ISS	US lab	0.132	0.101 (-24)	0.105 (-21)
		Cupola	0.115	0.102 (-12)	0.106 (-8)
Free space	Orion	HPU	0.336	0.345 (3)	0.346 (3)
		HSU1	0.328	0.340 (4)	0.340 (4)
		HSU2	0.320	0.330 (3)	0.329 (3)
	BioSentinel	BPD	0.276	0.287 (4)	0.282 (2)
Mars	Curiosity	MSLRAD	0.167	0.155 (-7)	0.147 (-12)

Values in parentheses are the relative difference (%) between model and measurement. Two sets of model calculations are provided. One set utilizes the native cross sections models developed for HZETRN2020, and the other set utilizes HZETRN2020 but with a coupled nucleon and pion interaction database generated from the Geant4 toolkit as described in the Methods section.

The magnitude of relative differences between model and measurement grows with increasing vertical cutoff rigidity, consistent with previous studies^14,20,21^. This systematic error originates from 3D scattering effects of low energy Compton electrons (<10 MeV) that are not well described by the HZETRN2020 deterministic methods. These electrons originate mainly from the electromagnetic cascade initiated by ultra-high energy (>5 GeV/n) nuclear collisions in which neutral pions are produced and rapidly decay to two photons (2γ). High energy nuclear interactions take place within shielding at all points along the ISS flightpath. However, the relative contribution of Compton electrons to total dose is enhanced at high cutoff rigidity, because the magnetosphere deflects a substantial fraction of the GCR that would otherwise contribute significantly to the dose-rate. It is important to recognize the systematic model error attributable to Compton electrons is only evident when the total absorbed dose-rate is smallest.

Data in Table 2 show that relative model errors for ISS are reduced in magnitude when the nucleon/pion Geant4 cross section databases are used. This is primarily a consequence of the simplified model used within HZETRN2020 to describe the angle and energy spectra of neutral pion production and rapid 2γ decay. The effect is more pronounced at high cutoff since, again, the magnetosphere deflects lower energy GCR ions, thereby enhancing the relative contribution to dose of secondary radiation produced from ultra-high energy interactions.

Relative model errors compared to measurements are larger in magnitude for the US Lab as compared to the Cupola. Although two different instruments are being considered, prior calibration and cross-validation studies have shown that total absorbed dose-rates measured by the Radiation Environment Monitor (REM) and Radiation Assessment Detector (RAD) instruments under the same radiation field conditions (i.e. co-located in the vehicle) are expected to agree within 5%, on average^22^. As shown in Table 3, the average dose-rate measured by RAD in the US Lab is ~15% larger than the REM measurement in the Cupola. Yet, the model results are quite similar between the two locations, as would be expected given the similarity in shielding distributions (see Fig. 4 and Table 1). This indicates that the discrepancy between the Cupola and US Lab locations is mainly attributable to dynamic and uncertain shielding configurations within the ISS that are not accounted for in the detailed CAD geometry models. Indeed, crewmembers move throughout the habitable volume and may move components or the detectors. These activities alter the mass shielding distribution surrounding the detector and influence measured exposures non-negligibly. Unfortunately, such information is nearly impossible to incorporate into CAD geometry models on a dynamic basis.

Model calculated dose-rates are in excellent agreement with measurements from the uncrewed Orion spacecraft and BioSentinel CubeSat. All model results were within 5% of the available free space measurements. In contrast to the situation on ISS where on-board mass and detectors may be moved inflight by crewmembers, the mass shielding geometry models and detector locations for Orion and BioSentinel were more precise and static throughout the Artemis-I mission timeframe. It is observed that both the measurements and model calculations are properly correlated with representative areal density values given in Table 1. For example, as the median thickness increases in moving from HSU2, to HSU1, to HPU, both the model calculations and measured dose-rates increase. Complexity and uncertainty associated with geomagnetic shielding is also avoided since the crafts were beyond the magnetosphere. The relative impact of low energy Compton electrons and neutral pion production and decay models is also significantly reduced in free space as compared to the high cutoff rigidity regions of the ISS trajectory. Correspondingly, the choice of nuclear model has negligible impact on the model results.

Finally, model results are within 7%–12% of the dose-rate measured on the surface of Mars by RAD during the Artemis-I mission. The choice of nuclear model has a moderate impact on model results—primarily due to differing nucleon and pion production multiplicities and energy/angle spectral characteristics. Although backscattered, or albedo, neutrons are known to be present on the Martian surface^17–19^ and are important for human risk estimation, they contribute little to the measured absorbed dose-rate in silicon being reported by the RAD detector. Hence, differences in diffuse neutron production between the nuclear models are likely not contributing to this case.

Additional factors influence the Martian surface radiation environment and may contribute to uncertainty in the calculations. The measured vertical atmospheric thickness of 21.4 g/cm^2^ was carried directly into the model evaluations, as seen in Fig. 4 and Table 1, and is not expected to be a significant source of uncertainty. The free space GCR environment used for model calculations is actually evaluated at 1 AU instead of 1.5 AU for Mars. However, data-driven heliospheric models suggest GCR intensities increase by at most a few percent in moving from 1 AU to 1.5 AU (Earth to Mars)^23^. Prior sensitivity tests^19^ have established that compositional details of the Martian atmosphere and regolith have little impact on surface dose-rates. The cylindrical geometry used to model the Martian atmosphere and surface is commonly used in such evaluations^17–19^, but nevertheless neglects topographical details (i.e. nearby boulders, hills, or valleys) and mass shielding that may influence measured and calculated surface dose-rates.

Broad consideration of the results in Table 3 shows that model calculations are within ~10–25% of ISS measurements, 4% of free space measurements, and 10% of Mars surface measurements. This level of agreement should be considered excellent given the overall model complexity, which includes: the natural GCR environment; geomagnetic shielding (for ISS in LEO); highly detailed mass shielding geometry for ISS, Orion, and BioSentinel; Martian atmospheric shielding; nuclear physics, and radiation transport.

### Context and outlook

Despite the apparently good agreement between model calculations and measurements, additional context and caution is needed for proper interpretation of the summary results. First, the exposure quantity of interest (absorbed dose-rate in silicon) and detectors considered herein are largely insensitive to neutrons. Model calculations also exhibited negligible direct contribution from neutrons. This is because all secondary charged particles produced in shielding by neutron interactions are explicitly transported in HZETRN2020. However, neutrons are particularly important from a human risk perspective^24,25^, given their high biological effectiveness and appreciable fluence behind thick shielding and on the lunar and Martian surfaces. Only limited spaceflight measurements of neutrons have been reported and have not covered the full energy range (1 MeV–1 GeV) of interest to human spaceflight risk^25^. Mass and power constraints severely limit the ability to measure neutrons >100 MeV inflight, and additional technology development is needed to resolve this gap.

Second, absorbed dose is an integrated quantity that can allow competing model errors to cancel, thereby obscuring potentially important uncertainties. This is particularly true in the case of space radiation, where the field contributing to absorbed dose comprises a complex mixture of particle types spanning many orders of magnitude in energy. Previous verification and validation efforts have demonstrated that while available models agree well with measurements (and each other) for absorbed dose, substantial disagreement can be found when underlying particle fluences are compared^8,17,18^. Such disagreements follow from gaps in ground-based experimental data for nuclear cross sections, particularly for production of pions, neutrons, and isotopes of hydrogen and helium^26^.

Finally, it remains true that accurately characterizing the space radiation environment encountered by humans is essential to assess exposure and subsequent health risks. However, the relationship between space radiation exposure and multiple adverse health consequences is highly uncertain. In the case of space radiation induced cancer mortality, risk projection models suffer from uncertainties exceeding 250%^11^. Additional sources of uncertainty, such as translating animal experimental data to humans, have yet to be characterized. Risk projections for cardiovascular disease or central nervous system decrements are even more uncertain due to limited (or complete lack of) terrestrial epidemiological data needed to foundationally support such models.

The collection of spaceflight radiation measurement devices and computational models appear highly capable to reliably calculate crew exposures for planned exploration missions. Continued research and development efforts are needed to address known gaps and uncertainties, particularly in the case of spaceflight neutron measurements and ground-based nuclear cross section measurements. However, the more substantial issue remains the vast uncertainties associated with relating crew exposure to subsequent health risks of interest. Clarifying dose-to-risk relationships is critical for providing accurate risk projections and communicating effectively to various stakeholders, include crewmembers, mission planners, and the public^3^. It is also needed to enable evaluation of various mitigation strategies, such as medical countermeasures, envisioned to play a key role in long duration exploration activities.

## Methods

### State-of-the-art models used to characterize crew exposure

The ambient GCR environment is described by the Badhwar-O’Neill model^27^ for the Artemis-I mission. The calculated spectra are used directly as the boundary condition for free space and Mars surface calculations. The LEO GCR environment is calculated by applying geomagnetic and terrestrial attenuation factors from the model of Badavi^28^ to the free space spectra.

Radiation transport codes are combined with nuclear and atomic physics models to describe how the ambient environment is modified as it passes through shielding and tissue. The NASA deterministic radiation transport code, referred to herein as HZETRN2020^12–14^, is based on perturbative solutions to the Boltzmann transport equation, resulting in highly efficient and accurate 3D numerical solutions. Verification studies have shown that HZETRN2020 in 3D mode can precisely reproduce Monte Carlo simulations provided the same nuclear interaction cross sections are used^14^.

HZETRN2020 utilizes a mixture of nuclear models to cover all energies and particles of interest to space radiation protection. Fragmentation of ions with mass, *A* > 4, is described by the RAADFRG model^29^; nucleon interactions are described by an intranuclear transport model^12,30^, and interactions involving pions, and isotopes of hydrogen and helium are treated within the DDFRG model^31,32^. Results were calculated using HZETRN2020 and this collection of nuclear physics models; additional calculations were performed with HZETRN2020 and coupled nucleon/pion cross section databases generated with Geant4^15^, as described elsewhere^14^.

The ISS and Orion geometries utilized in this study were developed in CAD software and contain over 11,000 and 23,000 parts, respectively. To couple such complex shielding geometries to HZETRN2020, ray-tracing methods are used to obtain material information surrounding a location of interest (i.e. detector location). Both vehicles were ray-traced at multiple detector locations with over 61,000 rays covering the unit sphere. From this ray-trace information, the shielding profile at any point within the volume can be accurately estimated^33^. Materials for each part were provided in the CAD models, although the overwhelming majority of parts were aluminum with unique densities. Sensitivity tests were completed to verify that redefining all parts to be made of aluminum had negligible impact on calculated exposures. Unique part densities were retained to ensure the overall geometric mass was preserved.

A combinatorial geometry description was used for BioSentinel^16^. It has a total mass of 14 kg and is a much simpler and smaller geometry compared to ISS (~400,000 kg) and Orion (~26,000 kg). The main geometry and solar panels were represented with nested and/or connected boxes. Materials for each box were defined from available information, although most of the primary payload mass consisted of aluminum, polycarbonate, stainless steel, and water. Solar panels were composed mainly of silicon. Densities of each box were set so that the known masses of major components were preserved.

Following prior verification and validation studies^17–19^, the geometry used to compute the Mars surface environment is defined as a series of stacked cylinders, with each cylinder having a radius of 829 km. The radius was chosen based on convergence tests aimed at retaining accuracy and minimizing computational cost. The bottom cylinder, representing the Martian surface, has a thickness of 4 m, and is composed of Martian regolith^19^. An additional 25 layers with equal areal densities (g/cm^2^) but varying volumetric density (g/cm^3^) and thickness (cm) are placed above the base regolith layer to represent the Martian atmosphere, which is composed of carbon dioxide and other trace elements^19^. The cumulative areal density of the 25 layers is set to 21.4 g/cm^2^—the value measured by the Curiosity REMS sensor during the time period of the Artemis-I mission. The density of each layer is modeled using the Mars Climate Database (MCD) 6.1^34,35^ under calm dust conditions. The location of the Curiosity rover at the time of the Artemis-I flight was used as input, and the solar longitude was calibrated such that the total areal density returned by the MCD model matched the measured value of 21.4 g/cm^2^.

### State-of-the-art instruments used to monitor intravehicular exposure levels

Accurate real-time measurements of the radiation field encountered by astronauts is critical for in-flight operations and post mission assessments. The real-time data may be used during solar storms to guide operational decisions and mitigation strategies^6^ and are also folded into acute biological response models^36,37^. During quiet-time (i.e. no active solar storm), the active dosimetry provides continuous monitoring of the GCR-induced radiation field at multiple intravehicular locations. Such data are combined with model assessments after the mission to ensure crew exposure and risk assessments are consistent with in-flight dosimetry^38^.

Two types of silicon-based detectors have been used in this study to measure absorbed dose-rates—Timepix and RAD. The Timepix detectors^39^ are 500 μm thick and consist of 256 × 256 pixels, with each pixel having a sensitive area of 3025 μm^2^ (55 μm × 55 μm). The effective sensitive area for the detector is 1.9825 cm^2^. They have been successfully flown on ISS^40^ for some time and were utilized on the Exploration Flight Test 1^41,42^ and BioSentinel missions^16^. They measured the absorbed dose for all particles depositing >5 keV. Extensive ground-based testing has also been completed at accelerator facilities using various radiation sources and particle beams of interest to spaceflight^43^ and each Timepix is individually calibrated and tested over a wide variety of dose rates at various accelerator beams. The Timepix detector flying on ISS is referred to as the REM.

The RAD instrument was first developed for the Mars Science Laboratory (MSL) as part of the Curiosity rover mission^44–46^. A modified version of MSL-RAD was developed for use on the ISS^47^, which provides greater power and mass allowances. Although the instruments are not identical, the feature used to measure the absorbed dose-rate in silicon on MSL-RAD and ISS-RAD are the same. RAD comprises three silicon detectors followed by CsI and plastic scintillators encapsulated by anti-coincidence shielding. For this paper, only data collected from the “B” silicon detector are used. The “B” silicon layer is a 300 μm thick planar detector with a surface area of 1.92 cm^2^. All particles that deposit energy above the detector threshold of ~30 keV contribute to the dose-rate measurement. It should be noted that for Mars surface measurements, Curiosity is powered by a radioisotope thermoelectric generator which makes a near-constant contribution to the dose rate in the “B” detector. This contribution has been estimated using data obtained in a test before launch, and a value corrected for the ~13% depletion of the ^238^Pu power source in the time since the test has been subtracted from the measured dose-rate^48^.

Absorbed dose-rates measured inside the ISS within the Cupola, Orion, and BioSentinel were obtained with Timepix detectors. Measurements inside the ISS within the US Lab and on the surface of Mars were obtained from the “B” silicon detector of the RAD instruments.

To isolate GCR contributions, the ISS measurement data were filtered^14,20,21,47^ to remove enhanced dose-rates caused by repeated orbital passes through the trapped proton belt within the SAA region (defined as locations where the magnetic field strength is <22.5 μT and the McIlwain L-shell value is <2). Both measurement data and modeling results for the ISS have been separated into vertical cutoff rigidity bins. Binning the ISS data in this way establishes the trend in dose-rates and model errors as the radiation environment in LEO approaches free space conditions^14,20,21^. The vertical cutoff rigidity is a model calculated value^28^ at any point along the ISS trajectory associated with the view direction pointing directly away from the Earth. Ions with rigidity below the vertical cutoff value are fully blocked by the magnetosphere. Vertical cutoff rigidity is loosely correlated with geographic latitude. The largest vertical cutoff rigidities are found at equatorial latitudes (i.e. strongest geomagnetic shielding), while smaller vertical cutoff rigidities are found approaching the poles (i.e. weakest geomagnetic shielding).

## Data Availability

All software and models used to calculate exposure quantities can be evaluated through the On-Line Tool for the Assessment of Radiation in Space (OLTARIS), available at https://oltaris.nasa.gov. Source code for many of the models can be downloaded through the NASA software repository at https://software.nasa.gov. MSL-RAD data are publicly available in the NASA Planetary Data System archive at https://pds-ppi.igpp.ucla.edu/collection/MSL-M-RAD-2-EDR-V1.0. The Timepix and ISS-RAD data will be placed on the publicly available the NASA RadLab website (https://visualization.osdr.nasa.gov/radlab/gui/overview) as soon as authorization is received, and until then, is available from authors at reasonable request.
